# Primary SARS-CoV-2 infection in patients with immune-mediated inflammatory diseases: long-term humoral immune responses and effects on disease activity

**DOI:** 10.1186/s12879-023-08298-6

**Published:** 2023-05-17

**Authors:** Koos P. J. van Dam, Adriaan G. Volkers, Luuk Wieske, Eileen W. Stalman, Laura Y. L. Kummer, Zoé L. E. van Kempen, Joep Killestein, Sander W. Tas, Laura Boekel, Gerrit J. Wolbink, Anneke J. van der Kooi, Joost Raaphorst, R. Bart Takkenberg, Geert R. A. M. D’Haens, Phyllis I. Spuls, Marcel W. Bekkenk, Annelie H. Musters, Nicoline F. Post, Angela L. Bosma, Marc L. Hilhorst, Yosta Vegting, Frederike J. Bemelman, Alexandre E. Voskuyl, Bo Broens, Agner Parra Sanchez, Cécile A. C. M. van Els, Jelle de Wit, Abraham Rutgers, Karina de Leeuw, Barbara Horváth, Jan J. G. M. Verschuuren, Annabel M. Ruiter, Lotte van Ouwerkerk, Diane van der Woude, Renée C. F. Allaart, Y. K. Onno Teng, Pieter van Paassen, Matthias H. Busch, Papay B. P. Jallah, Esther Brusse, Pieter A. van Doorn, Adája E. Baars, Dirk Jan Hijnen, Corine R. G. Schreurs, W. Ludo van der Pol, H. Stephan Goedee, Maurice Steenhuis, Sofie Keijzer, Jim B. D. Keijser, Olvi Cristianawati, Anja ten Brinke, Niels J. M. Verstegen, S. Marieke van Ham, Theo Rispens, Taco W. Kuijpers, Mark Löwenberg, Filip Eftimov, on behalf of the T2B! Immunity against SARS-CoV-2 study group

**Affiliations:** 1 Department of Neurology and Neurophysiology, Amsterdam Neuroscience, Amsterdam UMC, Location AMC, University of Amsterdam, Meibergdreef 9, 1105 AZ Amsterdam, the Netherlands; 2 Department of Gastroenterology and Hepatology, Location Academic Medical Center, Amsterdam UMC, University of Amsterdam, Amsterdam, the Netherlands; 3 Department of Clinical Neurophysiology, St Antonius Hospital, Nieuwegein, The Netherlands; 4 Department of Immunopathology, Sanquin Research and Landsteiner Laboratory, Amsterdam UMC, Amsterdam, the Netherlands; 5 Department of Neurology, Amsterdam UMC, VU University Medical Center, Amsterdam, the Netherlands; 6 Amsterdam Rheumatology and Immunology Center, Amsterdam UMC, Department of Rheumatology and Clinical Immunology, University of Amsterdam, Amsterdam, the Netherlands; 7 Amsterdam Rheumatology and Immunology Center, Location Reade, Department of Rheumatology, Amsterdam, the Netherlands; 8 Department of Dermatology, Amsterdam UMC, Location Academic Medical Center, University of Amsterdam, Amsterdam, the Netherlands; 9 Department of Internal Medicine, Section of Nephrology, Amsterdam UMC, Location Academic Medical Center, University of Amsterdam, Amsterdam, the Netherlands; 10 Department of Rheumatology and Clinical Immunology, Amsterdam Rheumatology and Immunology Center, VU University Medical Center, Amsterdam, the Netherlands; 11 Centre for Infectious Disease Control, National Institute for Public Health and the Environment (RIVM), Bilthoven, The Netherlands; 12 Faculty of Veterinary Medicine, Utrecht University Utrecht, Utrecht, The Netherlands; 13 Department of Rheumatology and Clinical Immunology, University Medical Center Groningen, Groningen, The Netherlands; 14 Department of Dermatology, Center for Blistering Diseases, University Medical Center Groningen, University Groningen, Groningen, The Netherlands; 15 Department of Neurology, Leiden University Medical Center, Leiden, the Netherlands; 16 Department of Rheumatology, Leiden University Medical Center, Leiden, the Netherlands; 17 Centre of Expertise for Lupus-, Vasculitis- and Complement-Mediated Systemic Diseases, Department of Internal Medicine – Nephrology section, Leiden University Medical Centre, Leiden, The Netherlands; 18 Department of Nephrology and Clinical Immunology, Maastricht University Medical Center, Maastricht, The Netherlands; 19 Department of Neurology, Erasmus MC University Medical Center, Rotterdam, the Netherlands; 20 Department of Dermatology, Erasmus MC University Medical Center, Rotterdam, the Netherlands; 21 Department of Neurology and Neurosurgery, Brain Center UMC Utrecht, Utrecht, the Netherlands; 22 Swammerdam Institute for Life Sciences, University of Amsterdam, Amsterdam, the Netherlands; 23 Department of Pediatric Immunology, Rheumatology and Infectious Disease, Amsterdam UMC, Location AMC, University of Amsterdam, Amsterdam, the Netherlands

**Keywords:** SARS-CoV-2, Covid-19, Autoimmune disease, Immune-mediated inflammatory diseases, Immunosuppression, TNF, Immunity, Antibodies, Disease activity, Flare

## Abstract

**Background:**

Patients with immune-mediated inflammatory diseases (IMIDs) on immunosuppressants (ISPs) may have impaired long-term humoral immune responses and increased disease activity after SARS-CoV-2 infection. We aimed to investigate long-term humoral immune responses against SARS-CoV-2 and increased disease activity after a primary SARS-CoV-2 infection in unvaccinated IMID patients on ISPs.

**Methods:**

IMID patients on active treatment with ISPs and controls (i.e. IMID patients not on ISP and healthy controls) with a confirmed SARS-CoV-2 infection before first vaccination were included from an ongoing prospective cohort study (T2B! study). Clinical data on infections and increased disease activity were registered using electronic surveys and health records. A serum sample was collected before first vaccination to measure SARS-CoV-2 anti-receptor-binding domain (RBD) antibodies.

**Results:**

In total, 193 IMID patients on ISP and 113 controls were included. Serum samples from 185 participants were available, with a median time of 173 days between infection and sample collection. The rate of seropositive IMID patients on ISPs was 78% compared to 100% in controls (*p* < 0.001). Seropositivity rates were lowest in patients on anti-CD20 (40.0%) and anti-tumor necrosis factor (TNF) agents (60.5%), as compared to other ISPs (*p* < 0.001 and *p* < 0.001, respectively). Increased disease activity after infection was reported by 68 of 260 patients (26.2%; 95% CI 21.2–31.8%), leading to ISP intensification in 6 out of these 68 patients (8.8%).

**Conclusion:**

IMID patients using ISPs showed reduced long-term humoral immune responses after primary SARS-CoV-2 infection, which was mainly attributed to treatment with anti-CD20 and anti-TNF agents. Increased disease activity after SARS-CoV-2 infection was reported commonly, but was mostly mild.

**Trial registration:**

NL74974.018.20, Trial ID: NL8900. Registered on 9 September 2020.

**Supplementary Information:**

The online version contains supplementary material available at 10.1186/s12879-023-08298-6.

## Background

Development of an adequate humoral immune response after an infection with severe acute respiratory syndrome coronavirus 2 (SARS-CoV-2) is an important mediator of protection against future infections [1]. Hybrid immunity (i.e., immunity in individuals who have been both vaccinated and infected) is superior to humoral immunity elicited by vaccination only [2]. Patients with immune-mediated inflammatory diseases (IMIDs) can have impaired humoral immune responses after SARS-CoV-2 vaccination, depending on the type of immunosuppressant (ISP) used [3]. However, data on humoral immune responses following SARS-CoV-2 infection are scarce and limited to specific diseases. Patients with inflammatory bowel diseases (IBD) treated with anti-tumor necrosis factor (TNF) and patients with multiple sclerosis (MS) or rheumatic disease receiving treatment with anti-CD20 therapies showed impaired humoral immune responses directly after infection [4–7]. The long-term humoral immune response following SARS-CoV-2 infections of patients with IMIDs should be studied in order to better understand the development of protective immunity in these patients.

Another concern is the possible interplay between infection and underlying disease activity in IMID patients. Previous studies indicated that a SARS-CoV-2 infection might trigger increased disease activity of the underlying IMID [8–11].

In this study we aimed to investigate long-term humoral immune responses and changes in disease activity after a primary SARS-CoV-2 infection in unvaccinated IMID patients using different types of ISPs.

## Methods

This is a substudy of an ongoing national multicenter observational cohort study in the Netherlands (Target-to-B! (T2B!) study), studying vaccination responses in IMID patients. For this substudy, we used baseline clinical and serological data at enrollment. A full description of the T2B! study with different types of IMIDs and ISPs that were included has been published previously [3].

We included IMID patients and healthy controls, with a SARS-CoV-2 infection any time before receiving the first SARS-CoV-2 vaccination (i.e. primary infection). A SARS-CoV-2 infection was defined as a positive PCR test or a positive antigen test. Suspected SARS-CoV-2 infections based solely on clinical symptoms were excluded. Participants who did not complete electronic questionnaires after enrollment were also excluded. Participants were recruited between February 2021 and July 2021. Data from this cohort has been used in previous studies [3, 7, 12–14].

Patients received electronic questionnaires at enrollment collecting clinical data on demographics, possible SARS-CoV-2 (re)infections before vaccination and whether an increase in disease activity occurred in the four weeks after infection. The investigators collected clinical data on IMID diagnosis, ISP use since January 2020, and coronavirus disease 2019 (COVID-19) severity (based on the WHO scale [15]) from patient files using an electronic case record form.

The date of SARS-CoV-2 infection was defined as the date of the PCR test or in case an infection was only proven by an antigen test, the date of symptom onset. PCR dates could be reliably retrieved by participants (for example from COVID-19 passports), whereas dates of antigen tests were not centrally registered and therefore usually not precisely known. Active treatment with ISPs was defined as receiving treatment during or in the three months before SARS-CoV-2 infection. ISPs with long-lasting biological effects (i.e. anti-CD20 therapy, cyclophosphamide, cladribine, and alemtuzumab) were also considered active treatment, if administered up to one year before infection.

Investigators extracted findings from patient files when a patient reported increased disease activity in the four weeks after infection, to assess whether the treating physician also reported an increased disease activity, and whether ISP treatment was intensified as a consequence of the increased disease activity.

A serum sample was collected by on-site venipuncture or home-based fingerprick, during enrolment before first vaccination against SARS-CoV-2, as described previously [3]. SARS-CoV-2 antibodies were measured using two different assays in a central laboratory. The primary assay to assess seropositivity was a semi-quantitative bridging enzyme-linked immuno sorbent assay (ELISA) measuring immunoglobulins of all isotypes against the receptor-binding domain (RBD) of the spike protein, which has a high sensitivity in very low antibody ranges (98·1% sensitivity and 99·5% specificity) [16]. In addition, we measured anti-RBD IgG titers (expressed in AU/ml) using a quantitative ELISA [17]. Results for anti-RBD antibodies were excluded if the sample arrived at the central laboratory more than 14 days after the first vaccination.

The primary outcome was the seropositivity rate for SARS-CoV-2 antibodies after primary SARS-CoV-2 infection. Seropositivity rates of specific groups of ISPs were considered a secondary outcome. Two specific ISP groups were formed a priori based on previous literature indicating impaired humoral immune responses after SARS-CoV-2 infection in IMID patients using ISPs (i.e., anti-CD20 therapy and anti-TNF therapy, both as mono- or combination therapy) [4–7]. IMID patients that were using ISPs other than anti-TNF or anti-CD20 agents were classified as ‘other ISPs’. Additional secondary outcomes were seropositivity rates and antibody titers in relation to time since infection. Due to a low number of observations, the anti-CD20 group was excluded from this analysis. Furthermore, the rate of patients with self-reported increased disease activity was documented, including the proportion of these patients in whom the physician reported increased disease activity and in whom treatment with ISPs was intensified.

Outcomes from patients with IMIDs on (any type of) ISP were compared to a combined control group of IMID patients not on ISPs and healthy controls (hereafter combined and named ‘controls’). The Wilcoxon rank-sum test and Kruskal–Wallis test were used to assess the difference in days between SARS-CoV-2 infection and sample collection between groups. For the difference in proportions between groups, Fisher’s exact test was used. Spearman’s rank correlation coefficient was used to test for correlation between time since infection and antibody titer. For the difference in antibody titers between two groups in time, Wilcoxon rank-sum test was used. Data analysis was performed by two authors (KPJvD and LW) using R version 4.2.1 (R foundation for Statistical Computing, Vienna, Austria).

## Results

**Fig. 1:**
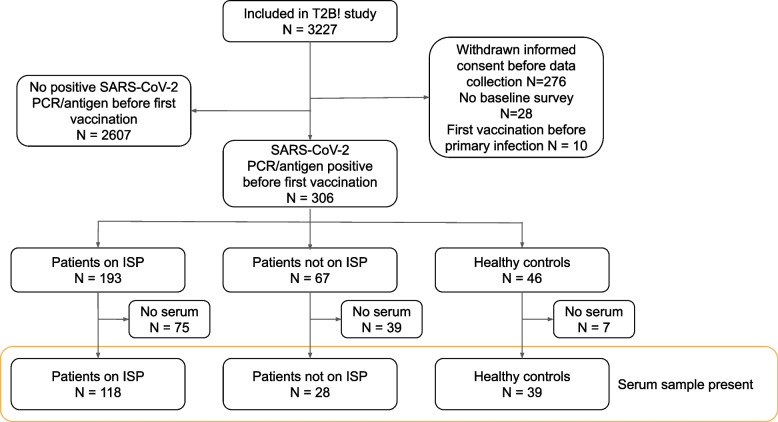
Study flowchart. *ISP* Immunosuppressant, *PCR* Polymerase chain reaction, *SARS-CoV-2* Severe acute respiratory syndrome coronavirus 2

In total, 306 participants with a primary SARS-CoV-2 infection were analyzed, including 193 IMID patients on ISPs, 67 IMID patients not on ISPs, and 46 healthy controls (Fig. 1). Participant characteristics are shown in Table 1. The mean age was 44.6 years (SD 13.8) and 192/306 (62.7%) participants were women. Focusing on patients using ISPs, 14/193 (7.3%) patients were on anti-CD20 agents (mono- or combination therapy)*,* 49/193 (25.4%) were on anti-TNF agents (mono- or combination therapy), and 130/193 (67.4%) used other ISPs (see Additional file 1, table S1 for detailed ISP treatments within groups). COVID-19 severity was classified as asymptomatic in 14/306 (4.6%) cases, mild in 279/306 (91.5%), moderate in 10/306 (3.3%) and severe (ICU admission) in 2/306 (0.7%). No participants died (Table 1).

**Table 1:** Participant characteristics. Table showing participant characteristics for patients with immune-mediated inflammatory disorder (IMIDs) treated with immunosuppressants (ISP), IMID patients without ISP and healthy controls

	**ISP (*N* = 193)**	**No ISP (*N* = 67)**	**Healthy controls (*N* = 46)**
**Age**			
Years, mean (SD)	43.8 (14.2)	46.2 (13.4)	45.6 (13.0)
**Sex, No (%)**			
Female	120 (62.2)	44 (65.7)	28 (60.9)
Male	73 (37.8)	23 (34.3)	18 (39.1)
**BMI**			
Mean (SD)	25.6 (4.6)	25.1 (4.4)	24.4 (3.4)
**IMID diagnosis, No (%)**			
* Rheumatic disease*			
Rheumatoid arthritis	25 (13.0)	0 (0)	NA
SLE	16 (8.3)	4 (6.0)	NA
Spondylarthritis	11 (5.7)	2 (3.0)	NA
Other rheumatic disease	4 (2.0)	1 (1.5)	NA
* Gastrointestinal disease*			
Crohn's disease	32 (16.6)	11 (16.4)	NA
Ulcerating colitis	19 (9.8)	3 (4.5)	NA
Other IBD	3 (1.6)	1 (1.5)	NA
* Neurological disease*			
MS and NMO	31 (16.1)	17 (25.4)	NA
Inflammatory neuropathies and myopathies	12 (6.2)	0 (0)	NA
Myasthenia gravis	11 (5.7)	3 (4.5)	NA
* Dermatological disease*			
Atopic dermatitis	12 (6.2)	0 (0)	-
Other dermatological	17 (8.8)	23 (34.3)	NA
* Other IMID*			
Other	0 (0)	2 (3.0)	NA
**Medication group, No (%)**			
Anti-CD20	14 (7.3)	0 (0)	0 (0)
Anti-TNF	49 (25.4)	0 (0)	0 (0)
Other ISP	130 (67.4)	0 (0)	0 (0)
**SARS-CoV-2 infection diagnosis, No (%)**			
PCR test positive	189 (97.9)	63 (94.0)	44 (95.7)
Antigen test positive	4 (2.1)	4 (6.0)	2 (4.4)
**COVID-19 severity, No (%)**^a^			
Asymptomatic	12 (6.2)	2 (3.0)	0 (0)
Mild	171 (88.6)	63 (94.0)	45 (100)
Moderate (hospitalization with oxygen)	5 (2.6)	1 (1.5)	0 (0)
Moderate (hospitalization without oxygen)	4 (2.1)	0 (0)	0 (0)
Severe (ICU admission)	1 (0.5)	1 (1.5)	0 (0)
Death	0 (0)	0 (0)	0 (0)
**Time from infection to sample**			
Days, median [IQR]	165 [96.3–224]	199 [90.5–233]	189 [85.5–230]

*BMI* Body mass index, *IBD* Inflammatory bowel disease, *ICU* Intensive-care unit, *IMID* Immune-mediated inflammatory disorder, *ISP* Immunosuppressant, *MS* Multiple sclerosis, *NMO* Neuromyelitis optica, *PCR* Polymerase chain reaction, *SARS-CoV-2* Severe acute respiratory syndrome coronavirus 2, *SLE* Systemic lupus erythematosus

^a^Missing due to withdrawn informed consent before COVID-19 severity could be assessed from medical records: 1/46 (2.2%) of healthy controls

**Fig. 2:**
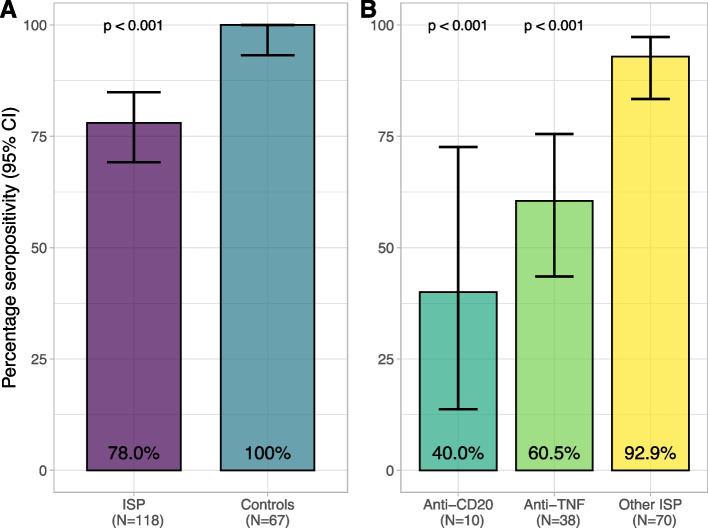
Seropositivity per specific ISP group. Seropositivity percentages of analyzed samples for **A**) ISP vs controls, **B**) specific ISP groups within the ISP group, with corresponding 95% CI’s. *CI* Confidence interval, *ISP* Immunosuppressant, *TNF* Tumor necrosis factor

Serum samples before vaccination were available for 190/306 (62.1%) participants. For five participants, serology results were excluded because the sample arrived more than 14 days after the first vaccination at the central laboratory, resulting in 185 samples that were analyzed (Fig. 1). From the analyzed samples, 57/185 (31%) were received at the laboratory before or at the day of first vaccination, 103/185 (56%) were received within the first week after first vaccination and 25/185 (14%) in the second week. No effect of time between first vaccination to arrival at the laboratory on the antibody titer was observed (Additional file 1, figure S1). The median time from infection to serum sample collection was 173 days [IQR 94.0–227], and this did not differ between ISP (165 days [IQR 96.3–224]) and controls (191 days [IQR 85.5–232], *p* = 0.45). Specific ISP groups were comparable in median time from infection to sample collection, with 155 days [IQR 146–185] for anti-CD20, 186 days [IQR 128–235] for anti-TNF, and 153 days [IQR 77.5–221] for other ISPs (*p* = 0.45). The rate of seropositive participants in IMID patients on ISPs (92/118; 78.0%), was lower compared to the control group (67/67; 100%; *p* < 0.001). A sensitivity analysis, restricting time from first vaccination to arrival at the laboratory to 7 days, did not change the results of the primary outcome (Additional file 1). From the patients on ISPs, patients on anti-CD20 were less often seropositive (4/10; 40.0%) compared to IMID patients on other ISPs (65/70; 92.9%; *p* < 0.001) or the control group (67/67; 100%; *p* < 0.001) (Fig. 2). In addition, patients on anti-TNF agents were less often seropositive (23/38; 60.5%) compared to IMID patients using other ISPs (65/70; 92.9%; *p* < 0.001) or the control group (67/67; 100%; *p* < 0.001). Seropositivity rates of patients on anti-CD20 therapy did not differ significantly from patients on anti-TNF agents (*p* = 0.30). The seropositivity rate of patients using anti-TNF monotherapy (12/22; 54.5%) was similar to patients using anti-TNF combination therapy (11/16; 68.8%; *p* = 0.51). A trend was found towards less seropositivity, but no significant difference was seen between IMID patients on other ISPs and controls (*p* = 0.06).

**Fig. 3:**
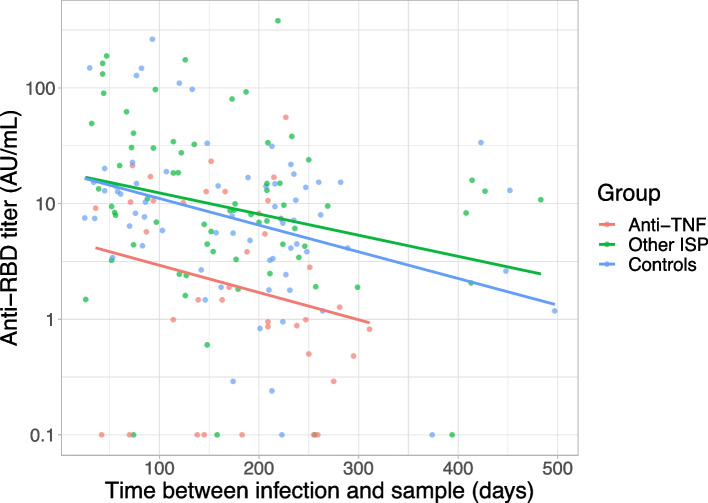
Effect of time since infection on anti-RBD antibody titer. Relation between SARS-CoV-2 RBD-specific antibody titer from all serum samples (dots) and time in days from SARS-CoV-2 infection to obtaining the serum sample. The colored lines are regression lines of titers, for patients with anti-TNF (r_s_ = -0.27; *p* = 0.05), other ISP (r_s_ = -0.29; *p* < 0.01), and controls (r_s_ = -0.32; *p* < 0.01). Colored dots reflect samples of patients with anti-TNF, other ISP, and controls. *AU* Arbitrary units, *ISP* Immunosuppressant, *RBD* Receptor-binding domain, *r*_*s*_ Spearman’s rho, *TNF* Tumor necrosis factor

Time between SARS-CoV-2 infection and sample collection showed a weak negative correlation with antibody titer for anti-TNF (*r*_s_ = -0.27 *p* = 0.05), other ISP (*r*_s_ = -0.29 *p* < 0.01), and controls (*r*_s_ = -0.32 *p* < 0.01); Fig. 3). The rate of seropositivity was compared for patients with an in infection in the six months before serum sample collection and an infection more than six months before serum sample collection, for patients on anti-TNF, other ISPs and controls (Additional file 1, figure S2). There was a non-significant trend towards less seropositivity in the anti-TNF group for patients with an infection more than six months before sample collection (10/19 52.6%) compared to an infection in the six months before sample collection (13/19 68.4%; *p* = 0.51). In comparison, all controls were seropositive in both time groups and proportions were comparable between the two time groups for the other ISP group.

**Fig. 4:**
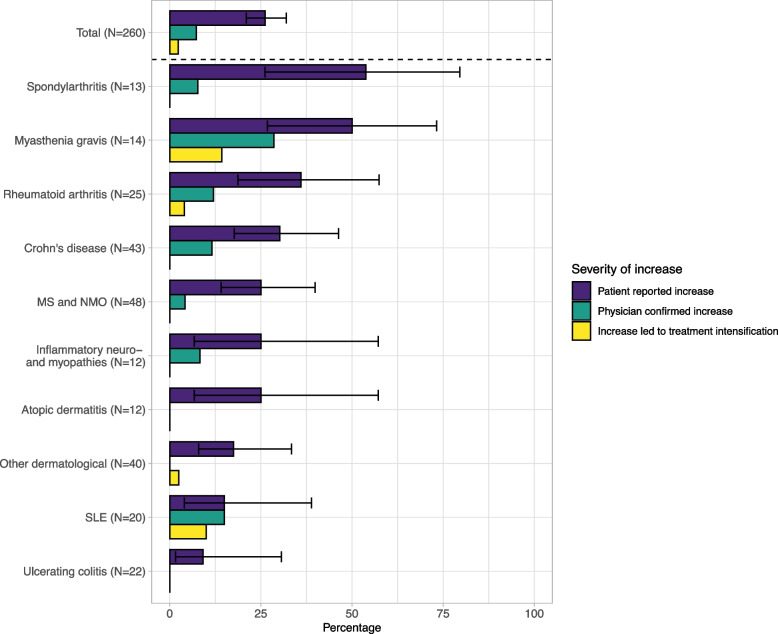
Disease activity after primary SARS-CoV-2 infection per IMID. Bar chart showing proportions of increase in IMID activity after SARS-CoV-2 primo infection per IMID. Different categories of severity of the increase in IMID activity are shown in colors. Proportions are all derived from the IMID group size, shown on the Y-axis. *IBD* Inflammatory bowel disease, *IMID* Immune-mediated inflammatory disorder, *MS* Multiple sclerosis, *NMO* Neuromyelitis optica, *SLE* Systemic lupus erythematosus

Of the IMID patients, 68/260 (26.2%; 95% CI 21.2–31.8%) reported increased disease activity within four weeks after SARS-CoV-2 infection. Self-reported increased disease activity was most frequently reported in patients with spondyloarthritis (7/13; 53.8%) and myasthenia gravis (7/14; 50.0%; Fig. 4). From all reported increases in disease activity, 19/68 (27.9%; 95% CI 18.7–39.6%) were also reported by the treating physician. The self-reported increased disease activity led to ISP treatment intensification in 6/68 cases (8.8%; 95% CI 6.1–21.5).

## Discussion

In this study, we found lower long-term seropositivity rates after SARS-CoV-2 infection in IMID patients on active treatment with ISPs compared to IMID patients who did not receive treatment with ISPs and healthy controls. This lower seropositivity rate was primarily explained by treatment with anti-CD20 or anti-TNF therapy. Finally, a quarter of patients reported increased disease activity after SARS-CoV-2 infection, leading to treatment intensification in a minority of patients.

The lower rate of seropositivity after a SARS-CoV-2 infection in patients using anti-CD20 therapy is in line with previous studies on the humoral immune response after SARS-CoV-2 infection, and our previous study on the humoral immune response after SARS-CoV-2 vaccination in patients on anti-CD20 [3, 7, 18]. Likewise, the decreased seropositivity rate in patients on anti-TNF therapy confirms previous studies that reported reduced humoral immune responses after SARS-CoV-2 infection in IBD patients on anti-TNF agents [5, 19]. However, these studies assessed antibodies against the nucleocapsid protein, which are unlikely to neutralize SARS-CoV-2 [20]. Studies assessing neutralizing antibodies against the RBD after SARS-CoV-2 infection in patients on anti-TNF are scarce. Boekel et al. reported no significant effect of anti-TNF on either seropositivity rate or anti-RBD antibody titers after infection compared to healthy controls, although the lack of effect may have resulted from selection bias towards seropositive participants [4]. To our knowledge, this is the first study demonstrating reduced anti-RBD antibody responses after PCR- or antigen-proven SARS-CoV-2 infection in patients using anti-TNF agents. Interestingly, early seropositivity rates after completed SARS-CoV-2 vaccination in our cohort and in other cohorts of IMID patients were not affected by anti-TNF, although titers were lower compared to healthy controls [3, 21]. Yet, multiple studies have shown a reduction in seropositivity rate, antibody titer and neutralizing capacity at six months after second vaccination in patients on anti-TNF [22–24]. Together, these results suggest that IMID patients on anti-TNF seem to be able to mount a relatively adequate humoral immune response, but also that this humoral response is less well maintained. In one study, it was hypothesized that anti-TNF leads to a reduced capacity to form de-novo long-lived SARS-CoV-2 specific plasma cells, which could explain the more rapid decay of antibodies over time [22]. For patients who used one of the other common prescribed ISPs, the seropositivity rate after SARS-CoV-2 infection was comparable to the control group of patients not using ISPs and healthy controls.

In line with previous work including different patient populations, the present study demonstrates that the antibody titer after SARS-CoV-2 infection decreases over time [2, 25, 26]. However, a significant number of IMID patients remained seropositive, even with a follow-up period exceeding six months. This indicates that there is considerable heterogeneity in long-term humoral immune responses among IMID patients on ISPs, and ISPs do not necessarily prevent long-term humoral immune responses.

Previous literature on increased underlying IMID activity after SARS-CoV-2 infection is largely limited to case reports and a few cohort studies [8, 11, 27]. Increased disease activity after a SARS-CoV-2 infection was reported in 41% of adult rheumatoid artritis patients. In a pediatric rheumatic cohort, a disease flare was reported in 16% of patients and another study found that 6% of patients suffering from systemic lupus erythematosus had a disease flare that was thought to be triggered by COVID-19. However, these studies used patient-reported disease activity only, without confirming severity of the increased disease activity based on reports of the physician and changes in treatment. In our cohort, increased disease activity in four weeks after SARS-CoV-2 infection was reported in more than a quarter of patients. In these patients, increased disease activity was also reported by physicians in one quarter, and resulted in ISP treatment intensification in a relatively small proportion of patients. This suggests that increased disease activity after infection is mild and mostly self-limiting, since treatment intensification was not often required. However, it is important to note that the COVID-19 severity in our study was generally classified as mild. In line with that notion, a mild infection may have a smaller chance of provoking an increase in disease activity compared to a severe infection due to less activation of the immune system. Interestingly, patients with spondyloarthritis and myasthenia gravis reported increased disease activity much more often than patients with other IMIDs, as nearly half of these patients reported increased disease activity. Infections are a well-known trigger of exacerbations in myasthenia gravis, whereas infections have not been found to be associated with increased disease activity in spondyloarthritis [28, 29]. Alternatively, COVID-19 symptoms such as myalgia and fatigue might mimic certain IMID symptoms, and might have contributed to the high proportion of patients with myasthenia and spondyloarthritis reporting increased disease activity. Moreover, reporting bias could have overestimated the incidence of increased disease activity after infection. Alltogether, it remains difficult to provide a true estimate of increased disease activity after infection nor to proof a causal relationship between COVID-19 and increased disease activity, especially in diseases where fluctuating symptoms are commonly seen.

The strengths of this study are the relatively large sample size of IMID patients with previous SARS-CoV-2 infections before vaccination, the disease overarching study-design and the control group. Compared to previous work, this study describes long-term humoral immune responses after SARS-CoV-2 infection, and therefore adds to the understanding of long-term humoral immunity in IMID patients on different types of ISP. Since participants were recruited up to August 2021, patients were either infected with the Wuhan, Alpha, Beta, Gamma or Delta SARS-CoV-2 strain. Meanwhile, measuring and interpreting long-term humoral responses is becoming increasingly difficult because of the interplay of different intervals between booster vaccinations, breakthrough infections with different SARS-CoV-2 variants and the resulting changing affinity of the humoral repertoire over time. Nonetheless, this study has some limitations. The samples arrived at the central laboratory up to 14 days after the first vaccination due to logistical reasons. Although patients were specifically instructed to perform a finger prick before vaccination, we cannot rule out that some samples were obtained after first vaccination, therefore possibly representing an early recall response. However, we did not observe an effect of time from first vaccination to arrival of the sample on the antibody titer, and a sensitivity analysis with a more restricted interval did not change the results of the primary outcome. Furthermore, the size of this cohort was not large enough to assess other specific groups of ISPs, which were shown to decrease seropositivity rates after SARS-CoV-2 vaccination, such as mycophenolate mofetil and S1P-modulators [3]. Also, SARS-CoV-2 antibodies were not measured longitudinally. Hence, we did not observe changes of the humoral immune response within individual participants. As we studied long-term antibody responses with low to very low antibody titers we relied on a semiquantitaive bridging ELISA to detect seropositivity and could therefore not reliably compare differences in antibody titers quantitatively between groups. Lastly, due to a small number of cases of increased disease activity in the different IMID groups, we could not study determinants of increased disease activity after infection or use disease-specific endpoints for increased disease activity.

## Conclusions

IMID patients on ISPs showed reduced long-term humoral immune responses after primary SARS-CoV-2 infection compared to participants not on ISPs. This difference could be mainly attributed to IMID patients treated with anti-CD20 and anti-TNF therapy. Overall, antibody titers decreased slightly over time, although most IMID patients on ISPs remained seropositive for more than six months following infection. Approximately a quarter of patients reported an increase in disease activity after SARS-CoV-2 infection, leading to ISP treatment intensification in only a minority of patients.

## Supplementary Information

**Additional file 1.**

## Data Availability

After publication, anonymized individual participant data and a data dictionary will be made available upon request to the corresponding author to researchers who provide a methodologically sound proposal. Data will be shared through a secure online platform.

## Abbreviations

AU: Arbitrary units
BMI: Body mass index
CI: Confidence interval
COVID-19: Coronavirus disease 2019
IBD: Inflammatory bowel diseases
ICU: Intensive-care unit
IMIDs: Immune-mediated inflammatory diseases
IQR: Interquartile range
ISPs: Immunosuppressants
MS: Multiple sclerosis
NMO: Neuromyelitis optica
PCR: Polymerase chain reaction
SARS-CoV-2: Severe acute respiratory syndrome coronavirus 2
SLE: Systemic lupus erythematosus
RBD: Receptor-binding domain
TNF: Tumor necrosis factor
