# Supplementary material for: Primary SARS-CoV-2 infection in patients with immune-mediated inflammatory diseases: long-term humoral immune responses and effects on disease activity
Source: BMC Infect Dis. 2023 May 17;23:332. doi: 10.1186/s12879-023-08298-6 (PMC10189216; doi:10.1186/s12879-023-08298-6)
Supplement: Supplementary file 1 — Additional file 1. [file 12879_2023_8298_MOESM1_ESM.docx]

**Additional file 1**

**Contents**

List of collaborators 2

Table S1: Detailed ISP treatments and 3

seropositivity within specific ISP groups

Figure S1 Effect of time from first vaccination 5

to sample arrival on antibody titer

Sensitivity analysis: results of primary outcome restricted to samples 6

received within seven days after first vaccination

Figure S2 Effect of time since 7

infection on seropositivity rate

**List of collaborators**

Rivka de Jongh - Sanquin, Amsterdam, The Netherlands

Carolien van de Sandt - Sanquin, Amsterdam, The Netherlands

Lisan Kuijper - Sanquin, Amsterdam, The Netherlands

Mariel Duurland - Sanquin, Amsterdam, The Netherlands

Ruth Hagen - Sanquin, Amsterdam, The Netherlands

Jet van den Dijssel - Sanquin, Amsterdam, The Netherlands

Christine Kreher - Sanquin, Amsterdam, The Netherlands

Amelie Bos - Sanquin, Amsterdam, The Netherlands

Veronique Konijn - Sanquin, Amsterdam, The Netherlands

Viriginia Palomares Cabeza - Sanquin, Amsterdam, The Netherlands

​​Sergey Nejentsev - Amsterdam UMC, Amsterdam, The Netherlands

Elham Mirfazeli - Amsterdam UMC, Amsterdam, The Netherlands

| **Table S1: Detailed ISP treatments and seropositivity within specific ISP groups**  Table showing detailed ISP treatment and seropositivity for participants in the specific ISP groups. |
| --- |

|  | **Number (% of specific ISP group)** | **Seropositivity (n/N, %)** |
| --- | --- | --- |
| **Anti-CD20 (N=14)** |  |  |
| Anti-CD20 monotherapy | 9 (64.3) | 3/6 (50.0) |
| Anti-CD20, corticosteroids | 2 (14.3) | 1/2 (50.0) |
| Anti-CD20, corticosteroids, immunoglobulin | 1 (7.1) | 0/0 (0) |
| Anti-CD20, corticosteroids, methotrexate | 1 (7.1) | 0/1 (0) |
| Anti-CD20, methotrexate | 1 (7.1) | 0/1 (0) |
| **Anti-TNF (N=49)** |  |  |
| TNF-alpha monotherapy | 28 (57.1) | 12/22 (54.5) |
| TNF-alpha, corticosteroids, | 1 (2.0) | 0/1 (0) |
| TNF-alpha, DHODH inhibitor | 1 (2.0) | 0/1 (0) |
| TNF-alpha, methotrexate | 10 (20.4) | 5/7 (71.4) |
| TNF-alpha, purine antagonist | 9 (18.4) | 6/7 (85.7) |
| **Other ISP (N=130)** |  |  |
| Belimumab, corticosteroids, mycophenolate mofetil | 1 (0.8) | 0/0 (0) |
| Calcineurin inhibitors monotherapy | 1 (0.8) | 1/1 (100) |
| Calcineurin inhibitors, corticosteroids | 1 (0.8) | 0/0 (0) |
| Calcineurin inhibitors, dupilumab | 1 (0.8) | 1/1 (100) |
| Corticosteroids monotherapy | 15 (11.5) | 5/5 (100) |
| Corticosteroids, immunoglobulin | 1 (0.8) | 1/1 (100) |
| Corticosteroids, immunoglobulin, methotrexate | 1 (0.8) | 0/0 (0) |
| Corticosteroids, methotrexate | 1 (0.8) | 1/1 (100) |
| Corticosteroids, methotrexate, tocilizumab | 1 (0.8) | 0/0 (0) |
| Corticosteroids, mycophenolate mofetil | 2 (1.5) | 1/1 (100) |
| Corticosteroids, purine antagonist | 8 (6.2) | 2/3 (66.6) |
| DHODH inhibitor monotherapy | 2 (1.5) | 0/1 (0) |
| Dimethylfumarate monotherapy | 8 (6.2) | 5/5 (100) |
| Dupilumab monotherapy | 11 (8.5) | 6/7 (85.7) |
| Hydroxychloroquine monotherapy | 9 (6.9) | 5/5 (100) |
| IL-17A antagonist monotherapy | 1 (0.8) | 1/1 (100) |
| Immunoglobulin monotherapy | 6 (4.6) | 2/2 (100) |
| JAK inhibitor monotherapy | 3 (2.3) | 2/2 (100) |
| Methotrexate monotherapy | 17 (13.1) | 12/12 (100) |
| Methotrexate, vedolizumab | 1 (0.8) | 1/1 (100) |
| Mycophenolate mofetil monotherapy | 3 (2.3) | 3/3 (100) |
| Natalizumab monotherapy | 8 (6.2) | 3/4 (75) |
| Purine antagonist monotherapy | 17 (13.1) | 8/9 (88.9) |
| S1P-receptor modulator monotherapy | 3 (2.3) | 1/1 (100) |
| Ustekinumab monotherapy | 4 (3.1) | 2/2 (100) |
| Ustekinumab, vedolizumab | 1 (0.8) | 1/1 (100) |
| Vedolizumab monotherapy | 3 (2.3) | 1/1 (100) |

DHODH: dihydroorotate dehydrogenase; IL: interleukin; IMID: immune-mediated inflammatory disease; ISP: immunosuppressant; JAK: janus kinase; S1P: sphingosine 1-phosphate; TNF: tumor necrosis factor.

**Figure S1 Effect of time from first vaccination to sample arrival on antibody titer**

Antibody titers of participants individual participants, stratified by time from infection to sample arrival (top) and time from first vaccination to sample arrival (bottom). Antibody titer did not differ for the different times from first vaccination to sample arrival for the other ISP group with time from infection to sample arrival <180 days (p=0.50; Kruskal Wallis test between all timepoints), for the control group with time from infection to sample arrival <180 days (p=0.15; Kruskal Wallis test between all timepoints), for the other ISP group with time from infection to sample arrival ≥180 days (p=0.98; Kruskal Wallis test between all timepoints), and for the control group with time from infection to sample arrival ≥180 days (p=0.76; Kruskal Wallis test between all timepoints).

**
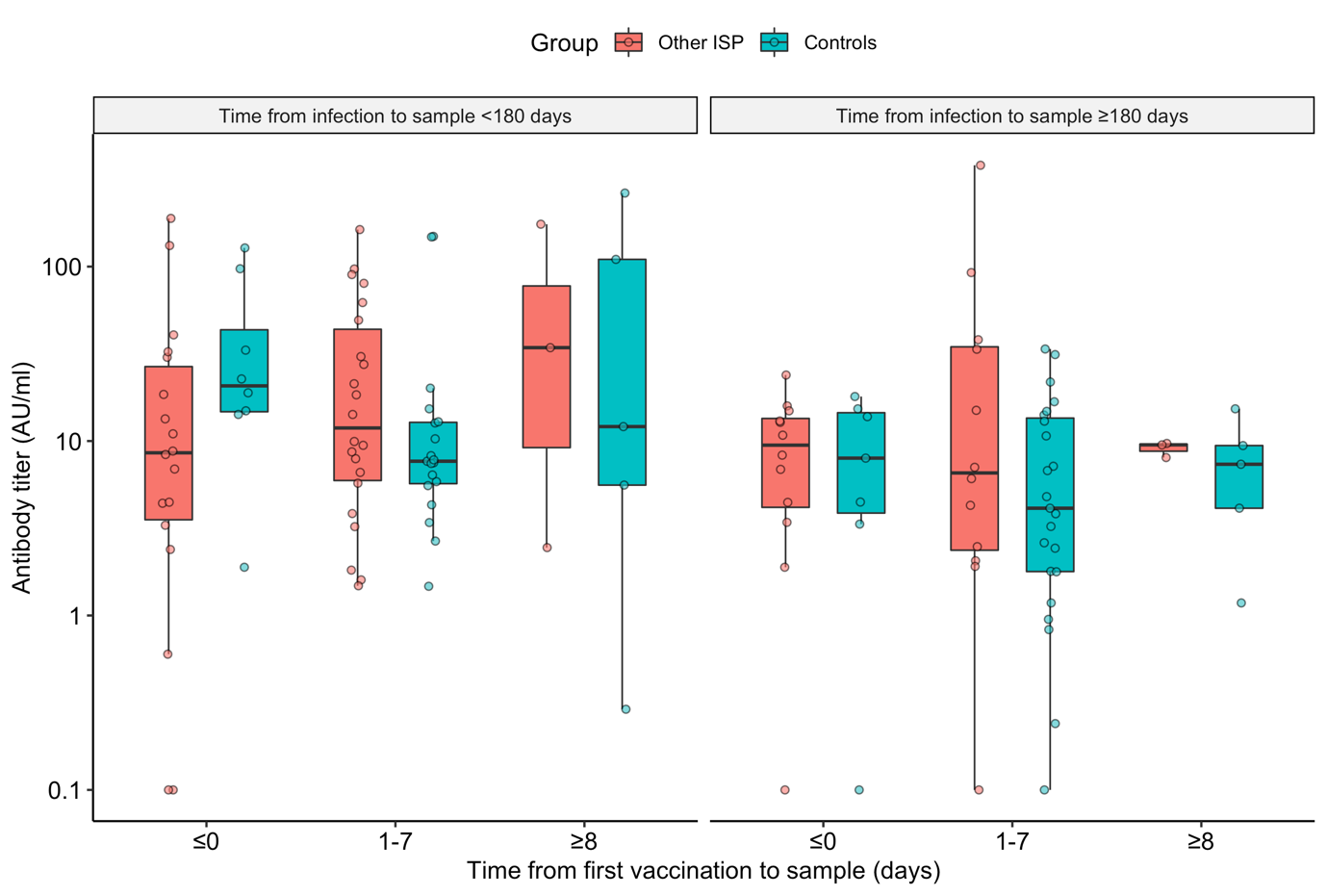
**

AU: arbitrary units; ISP: immunosuppressants

**Sensitivity analysis: results of primary outcome restricted to samples received within seven days after first vaccination**

To assess if samples received in the second week after vaccination influenced our findings, because a vaccination induced humoral response might already be present, we repeated the analysis of the primary outcome on samples that arrived at the central laboratory within seven days after first vaccination. For this analysis, 160 samples were included. The rate of seropositive participants in IMID patients on ISPs (84/103; 81.6%), was lower compared to the control group (57/57; 100%; p<0.01). These proportions are comparable to the results of the primary analysis, where seropositivity in ISPs is 92/118 (78.0%) and in the control group 67/67 (100%; p<0.01).

**Figure S2 Effect of time since infection on seropositivity rate**

Seropositivity percentages of analyzed samples with corresponding 95% CI’s for (PCR and/or antigen proven) SARS-CoV-2 infection occurring 0-6 or >6 months prior to sampling for patients with anti-TNF, other ISP, no ISP and healthy controls.

CI: confidence interval; ISP: immunosuppressants; PCR: polymerase chain reaction; TNF: tumor necrosis factor
